# Microalbuminuria and obesity in children

**DOI:** 10.1186/1687-9856-2013-S1-P93

**Published:** 2013-10-03

**Authors:** Byung Ok Kwak, Hye Won Park, Hyung Jung Kim, Sochung Chung, Kyo Sun Kim

**Affiliations:** 1 Department of Pediatrics, Konkuk University Medical Center, Seoul, Korea; 2 Department of Pediatrics, Konkuk University School of Medicine, Seoul, Korea

## 

Microalbuminuria is considered as a marker of endothelial dysfunction and found to be associated with nephropathy and cardiomyopathy in diabetic and nondiabetic obese patients. In this study, we sought to investigate the effect of childhood obesity on microalbuminuria in children. A total of 96 children who were clinically healthy were enrolled from July 2007 through March 2012. Height, weight and body composition of fat mass (FM) and fat free mass (FFM) were measured in each subject. Body mass index (BMI), fat mass index (FMI), fat free mass index (FFMI) and percent body fat (PBF) were calculated. Serum creatinine, cholesterol, triglyceride (TG), high-density lipoprotein (HDL) cholesterol, fasting insulin, glucose, spot urine microalbumin/creatinine ratio, and glomerular filtration rate (GFR) were obtained. We divided the children into two groups according to the presence of microalbuminuria, based on the age specific reference range of spot urine microalbumin/creatinine ratio(1) and the differences between two groups were analyzed. Urine creatinine and FFMI in microalbuminuria group were found to be significantly decreased compared to control group. There were no significant differences between two groups in anthropometric and other biochemical parameters associated with obesity. In two groups comparison according to BMI z-score: lean (BMI z-score <2) and obese (BMI z-score ≥2), obese children showed significantly higher serum creatinine, TG, FM, PBF and FMI and lower HDL cholesterol. However, no significant differences in the urinary microalbumin excretion and FFMI were observed. In our study, urinary microalbumin excretion is associated with FFMI rather than obesity in children. This suggests that microalbuminuria might be strongly associated with FFM rather than obesity during growth. Further studies to elucidate the implication of microalbumiuria in obese children should be followed.

